# Sir2 plays a key role in cell fate determination upon SAPK activation

**DOI:** 10.18632/aging.100419

**Published:** 2011-12-30

**Authors:** Alexandre Vendrell, Francesc Posas

**Affiliations:** Cell Signaling Unit, Departament de Ciències Experimentals i de la Salut, Universitat Pompeu Fabra (UPF), E-08003, Barcelona, Spain

**Keywords:** Sirtuins, Sir2, SAPK, yeast, stress, apoptosis, p38

## Abstract

Although the benefit of sirtuin activation in age-related diseases is well-characterized, the benefit of sirtuin activation in acute diseases has been elusive. Here we discuss that, at least in yeast, Sir2 activation prevents programmed cell death induced by the sustained activation of the stress activated protein kinase (SAPK) Hog1, the yeast homologue of the p38 SAPK. Sir2 prevents ROS formation and maximize cell survival upon SAPK activation. The conserved function of Sir2 in age-related diseases and the conserved role of SAPKs open the possibility of a novel role for sirtuins in cell fate determination in eukaryotic cells.

## INTRODUCTION

Activation of sirtuins has been proved to be positive in age-related diseases [1-5]. However, the role of sirtuin activation in acute diseases, such as inflammatory processes, has not been clearly defined. Stress-activated protein kinases (SAPKs) are essential components for intracellular signalling networks that serve to respond and adapt to extracellular changes. Stress-activated protein kinases play a key role in signal transduction to extracellular insults and have been involved in numerous diseases, including inflammatory processes [6-8]. Exposure of yeast to high osmolarity results in the transient activation of p38-related SAPK, Hog1, the yeast homologue of the p38 SAPK [9, 10]. The transient activation of the SAPK serves to control many aspects of the cell physiology ranging from control of metabolism, cell cycle progression and gene expression which are essential for cellular adaptation to stress [11-15]. Despite the essential role of Hog1 in cell survival upon stress, it has been known for a long time that sustained activation of the SAPK was deleterious for cell growth as it has been shown by other MAPKs [16-18]. Therefore, the dynamics of Hog1 activation are critical for cell fate determination. However, the mechanism by which Hog1 was deleterious for cell growth remained unknown.

**Figure 1:**
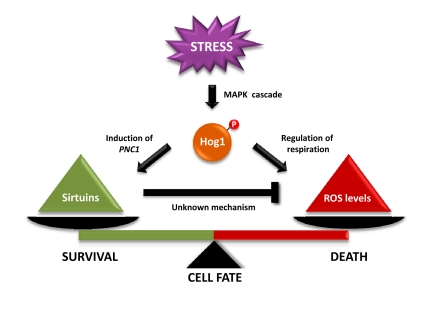
Tentative model that depicts the effect of Hog1 and sirtuins in dictating cell-fate determination. Hog1 inhibits mitochondrial respiration, which results in an increase in reactive oxygen species (ROS) accumulation that leads to cell death. In parallel, Hog1 induces *PNC1* expression. Pnc1 activates Sir2, which mediates a decrease in ROS accumulation. Sir2 activation by the stress-activated protein kinase Hog1 relieves the Hog1-induced oxidative stress to prevent apoptotic cell death.

The study by Vendrell et al shows that inhibition of cell growth by sustained activation of Hog1 is caused by the induction of apoptosis-like cell death in yeast. Specifically, activation of the Hog1 SAPK inhibits mitochondrial respiration that leads to an increase in ROS levels (Figure 1). This increase in ROS levels correlates with a decrease in mitochondrial respiration and it is prevented when cells are grown in the absence of oxygen. Therefore, the increase in ROS production seems to be caused by electron transport inhibition.

Several apoptotic makers can be detected upon Hog1 activation; such as an increase in SubG1 cells, DNA aggregation, TUNEL assay positive cells and metascaspase activation. Remarkably, deletion of genes encoding apoptosis mediators, such as the Omi yeast homologue (NMA111) or, to a lesser extent, the YCA1 caspase prevented Hog1-induced cell death. Taken together, Hog1-induced cell death is likely to be caused by apoptosis.

**Figure 2:**
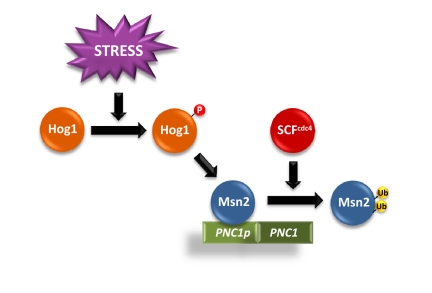
Scheme of Hog1-induced Msn2/Msn4 dependent gene expression upon SCF^cdc4^ regulation. Activation of Hog1 SAPK induces the activation of Msn2/Msn4-dependent genes, such as *PNC1*. The E3 _SCF_cdc4 ubiquitin ligase ubiquitylates the transcription factors to promote their degradation. Partial inactivation of SCF^cdc4^ decreases Msn2/4 degradation, which results in an increased accumulation of the TFs at the promoters and to an increase in Msn2/Msn4 dependent gene expression.

Unexpectedly, mutations in the E3 ubiquitin ligase CDC4, as well as the core components of the SCF complex (SKP1 and CDC53) partially suppressed Hog1-induced cell death. SCF mutations resulted in a reduced Msn2/Msn4 degradation that led to an increase in Msn2/Msn4 dependent gene expression (Figure 2).

Interestingly, PNC1, an activator of the Sir2 histone deacetylase, is under the control of Msn2/Msn4 transcription factors [19]. The mutations on SCFcdc4 resulted in an increased association of Msn2/4 at the PNC1 promoter and to an increased PNC1 expression. The relevance of Msn2-mediated PNC1 expression and the role of Sir2 in cell survival are illustrated by the fact that deletion of MSN2/4, PNC1 or SIR2 resulted in cells unable to suppress the apoptosis caused by sustained activation of Hog1. In contrast, over-expression of MSN2, PNC1 or SIR2 prevented Hog1-induced cell death. Therefore, Sir2 activation is important to prevent SAPK-induced apoptotic cell death (Figure 1).

The mechanism by which Sir2 prevents apoptosis in yeast has not been defined. However, whereas Sir2 activation does not alter cellular respiration upon stress, it strongly reduces ROS production and hence, it is likely that this reduction in ROS permits cell survival upon Hog1 activation.

Activation of SAPK signalling is essential for cell adaptation to stress. Strikingly, sustained activation of the pathway unravels a more-complex scenario. Therefore, although a decrease in mitochondrial respiration might be important for adaptation to stress, an extended reduction of respiration leads to excessive ROS formation. To counteract cell damage, Hog1 seems to engage a protective program by inducing PNC1 gene expression, which concomitantly activates Sir2 to balance excessive ROS accumulation. Therefore, cell fate is dictated by the balance between ROS induced by Hog1 SAPK and the protective effects of Sir2.

## DISCUSSION

### MAPKs, apoptosis and mitochondrial function

Apoptosis is caused by a variety of factors, such as oxidative stress [20]. Induction of apoptosis in response to MAPK activation has been described in several cell types and organisms [17, 21-24]. The p38 MAPK is activated by several stresses as well as oxidative stress [25, 26]. However, it is not known whether p38 is able to cause oxidative stress by altering mitochondrial function. Data from yeast suggests that, directly or indirectly, MAPKs can modulate mitochondrial respiration and ROS production. Then, it would be interesting to test whether, once p38 is activated, the MAPK regulates mitochondrial activity and ROS levels. A putative scenario would be that an increase of ROS could serve to create a positive feedback loop to sustain MAPK activation in the presence of stress.

The mechanism by which Hog1 regulates mitochondrial respiration and ROS levels remains unknown. It cannot be excluded that regulation of metabolism could lead to an altered TCA cycle and/or mitochondrial respiration. Therefore, the characterization of this phenomenon will allow identifying at which level the MAPKs can interact with the mitochondria: regulating mitochondria biogenesis, respiratory chain formation or respiratory chain activity. This would help to know whether the effect of Hog1 MAPK could be conserved in higher eukaryotes.

### Sirtuins, apoptosis and lifespan extension

The effect of sirtuins in apoptotic cell death is not clear. Depending on expression of a specific sirtuin, the cell type and the stimuli, sirtuins prevent or induce apoptosis [27-29]. Moreover, it is not clear whether the effect of sirtuin overexpression to extend lifespan in higher eukaryotes, such as worms and flies [30]. In yeast, Sir2 prevents apoptosis mediated by Hog1. There have been several molecular alterations associated to replicative ageing, such as the accumulation of extrachromosomal ribosomal DNA circles (ERCs), the assimetrically distribution of oxidatively damaged proteins and/or altered mitochondria that seems to be, at least partially controlled by Sir2 [31-35]. However, the relationship of these phenomena and the induction of cell death is not yet defined. In yeast, Sir2 effect upon Hog1 activation Net1 (an anchor for Sir2 to rDNA) but not on Sir4 or HM loci [31, 36, 37]. This suggests that the role of Sir2 in Hog1-mediated cell death might be associated to ERCs in contrast to other Sir2 functions.

Recent data on the overexpression of mammalian Sir2 seems to show that Sir2 might not be responsible of lifespan extension albeit important for health in ageing processes [38]. Remarkably, overexpression of Sir2 prevents apoptosis in yeast. It is worth noting that this preventive effect is only observed upon strong overexpression of Sir2. Two-fold overexpression of Sir2 did not show a significant effect on preventing apoptosis upon Hog1 sustained activation. A recent study in mice with moderately overexpression of Sirt1 reports prevention in age-related diseases, but without effects in lifespan [5]. Therefore, the degree of Sir2 expression might be critical to affect some age-related effects that might be associated to the degree of oxidative stress in the cell.

### Activation and regulation of apoptosis in an unicellular model

Apoptosis in yeast is a controversial issue. The benefit that could generate programmed cell death in a unicellular organism has been deeply discussed [39]. It is thought that the benefit of programmed cell death in yeast might be associated to a population rather to an individual [40]. Upon stress, the older or damaged individuals could enter into programmed cell death to allow other cells, genetically identical to them, increase their resources to survive.

The determination of cell fate upon stress could result of the balance between cell survival and cell death. Once cells are exposed to a stress, Hog1 SAPK is activated and cells enter into an alarm state. Upon a mild stress, Hog1 SAPK is active for a short period of time. Then, Hog1-induced sirtuin activation might be sufficient to prevent ROS accumulation, and the whole population survives. However, upon severe stress or sustained activation of the SAPK, longer activation of the MAPK Hog1 induces an increase on ROS production beyond a certain threshold. It is likely then that the preventive effect of sirtuin activation might not be sufficient to compensate ROS levels and certain cells of the population would undergo apoptosis and provide more resources to the younger cells to adapt and survive to a severe stress. The fact that both pro and anti-apoptotic pathways are controlled by the same protein, Hog1, emphasize the importance of the balance between the two responses that will determine cell fate.

## FUTURE DIRECTIONS

Vendrell and collaborators have unraveled the ability of Sir2 to prevent ROS formation and cell death upon MAPK activation in yeast. However, the mechanism by which Sir2 prevents ROS formation remains unclear. It would be interesting in the field of sirtuins to define how Sir2 is able to reduce ROS levels and decipher whether Sir2 is modulating mitochondrial activity per se or it is inducing antioxidant defence mechanisms.

It is likely that, as occurs in yeast, the balance in sirtuin activation determines cell fate in higher eukaryotes cells. Recent data show that two-fold overexpression of Sir2 in flies and worms did not extend lifespan at least as much as it was reported before [41-45]. In fact, other previous works showed that, also in yeast, two-fold Sir2 overexpression does not extend lifespan in some backgrounds [46]. Data provided by Vendrell et al show the importance of sirtuin dose and activation in preventing ROS accumulation and apoptosis in yeast. Since only dramatic overexpression of Sir2 is able to prevent apoptosis in yeast, further studies with stronger sirtuin overexpression might uncover the role of sirtuins in higher eukaryotes.
